# Complete Mitochondrial Genome of the Citrus Spiny Whitefly *Aleurocanthus spiniferus* (Quaintance) (Hemiptera: Aleyrodidae): Implications for the Phylogeny of Whiteflies

**DOI:** 10.1371/journal.pone.0161385

**Published:** 2016-08-23

**Authors:** Zhi-Teng Chen, Li-Xia Mu, Ji-Rui Wang, Yu-Zhou Du

**Affiliations:** 1 School of Horticulture Plant Protection & Institute of Applied Entomology, Yangzhou University, Yangzhou, 225009, China; 2 School of Agricultural & Food Science, Zhejiang Agriculture & Forestry University, Linan, 311300, China; INRA, FRANCE

## Abstract

In this study, we sequenced the complete mitochondrial genome (15,220 bp) of the citrus spiny whitefly, *Aleurocanthus spiniferus* (Quaintance), a well-known pest from the superfamily Aleyrodidae. The *A*. *spiniferus* mitogenome contains 36 genes, including 13 protein-coding genes (PCGs), 21 transfer RNAs (tRNA), two ribosomal RNAs (rRNA) and a large non-coding region (control region, CR). Like most whiteflies, the *A*. *spiniferus* mitogenome had a large degree of rearrangement due to translocation of the *nad3-trnG-cox3* gene cluster. The 13 PCGs initiated with ATN and generally terminated with TAA, although some used TAG or T as stop codons; *atp6* showed the highest evolutionary rate, whereas *cox2* appeared to have the lowest rate. The *A*. *spiniferus* mitogenome had 21 tRNAs with a typical cloverleaf secondary structure composed of four arms. Modeling of the two rRNA genes indicated that their secondary structure was similar to that of other insects. The CR of *A*. *spiniferus* was 920 bp and mapped between the *nad3-trnG-cox3* and *trnI-trnM* gene clusters. One potential stem-loop structure and five tandem repeats were identified in the CR. Phylogenetic relationships of 11 species from the Aleyrodidae were analyzed based on the deduced amino acid sequences of the 13 PCGs and evolutionary characteristics were explored. Species with more genetic rearrangements were generally more evolved within the Aleyrodidae.

## Introduction

*Aleurocanthus spiniferus* (Quaintance), the citrus (orange) spiny whitefly (Hemiptera: Aleyrodidae), is an important pest of citrus (*Citrus* spp.; Rutaceae) and tea (*Camelia sinensis* (L.) Kuntze; Ericales: Theaceae). *A*. *spiniferus* is indigenous to tropical Asia and was first reported in Japan [1]; thereafter, it spread to Africa, Australia, America, the Pacific Islands and Italy [2–3] and became a highly destructive pest [4]. There are two main types of damage caused by *A*. *spiniferus*; the first type is the direct damage caused when immature and adult whiteflies ingest phloem sap, which leads to a weakened host plant and premature senescence. The second type of damage is caused when the whiteflies excrete honeydew on leaf surfaces; this promotes the growth of sooty mold, interferes with photosynthesis, and reduces plant quality [5]. In addition, numerous puparia of *A*. *spiniferus* may reside on the abaxial surface of infested leaves, which provides a safe overwintering site for a variety of mite pests [6]. In addition to citrus and tea, *A*. *spiniferus* infests many other economically important plants including rose, grape, peach, pear, and guava [7]. It is important to mention that *A*. *spiniferus* is on the list of quarantined species published by EPPO and was recently moved from the A1 to the A2 list [8]. In recent years, mitogenome has become an informative molecular structure widely used in studies focusing on species identification, phylogeography, evolutionary biology, molecular evolution, phylogenetic inference, and population genetics [9–13]. Generally, animal mitogenomes possess many unique characteristics, including maternal inheritance, lack of intermolecular recombination, and faster evolutionary frequency than nuclear DNA [14–15]. Thao et al. [13] sequenced the mitogenomes of six whitefly, one aphid, and one psyllid species and discovered that four had rearrangements in the *cox3-nad3* region as compared to the hypothesized model insect mitogenome. Based on these rearrangements, they speculated that this region underwent a transposition and at least four types of gene rearrangement had occurred in the evolution of whiteflies. Although the genes contained in the insect mitogenome are highly conserved, rearrangements or even disappearance of these 37 genes often occur, and the position of tRNA genes rearrange more frequently than the PCGs and rRNA genes in some species [16].

Currently, many aspects of *A*. *spiniferus*, including the biology, behavior, ecology, and management have been thoroughly investigated [17–22]. However, complete mitogenomic data for *A*. *spiniferus* have been lacking; consequently, the phylogenetic position of this species is unclear [23–24]. Furthermore, determination of the mitogenome of the citrus whitefly will contribute to our understanding of whitefly mitogenome structures and phylogenetics in the Hemiptera. In this study, we sequenced the complete mitogenome of *A*. *spiniferus* and provided a thorough description of its genome features. Finally, we discussed phylogenetic relationships and evolutionary traits among species of the Aleyrodidae based on the sequenced mitogenomes.

## Materials and Methods

### Sample preparation and DNA extraction

Adult specimens of *A*. *spiniferus* were collected at West Lake (30°15'01.97"N, 120°09'44.91"E) in September, 2012, Zhejiang Province, China. All studies were conducted on public lands; our research activities were not banned by any organization or individual and did not involve endangered or protected species. Samples were identified, preserved in 100% ethanol, and stored at −20°C until DNA was extracted. Whole genomic DNA was extracted from individual samples using DNAVzol (Bioteke, Beijing, China) and stored at −20°C until used for PCR.

### PCR amplification, cloning and sequencing

The mitogenome of *A*. *spiniferus* was amplified with 15–20 overlapping PCR fragments. PCR primers were designed using Primer Premier 5.0 software and were based on universal primers of insect mitogenome [14] and 11 Sternorrhyncha mitogenome sequences that were available in GenBank [13] (Table A in S1 File).

Conditions for PCR amplification were as follows: initial denaturation for 5 min at 94°C, followed by 35 cycles at 94°C (30 s each), annealing for 1 min at 45–60°C, elongation for 1–3 min (depending on putative length of the fragments) at 72°C, and a final extension step of 10 min at 72°C. LA Taq polymerase (TaKaRa, Dalian, China) was used for PCR amplification, except for fragments less than 1.2 kb, which were amplified with Taq polymerase (TaKaRa). All PCR reactions were performed in an ABI thermal cycler (PE Applied Biosystems, San Francisco, CA, USA). PCR products were separated by 1.0% agarose gel electrophoresis. Purified PCR products were ligated into pGEM-T Easy Vector (Promega). Recombinant clones were sequenced in both directions using the BigDye Terminator Sequencing Kit (Applied BioSystems) and the ABI 3730XL Genetic Analyzer (PE Applied Biosystems, San Francisco, CA, USA) with vector-specific and internal primers. All PCR fragments were sequenced after separation and purification.

### Sequence, analysis and secondary structure prediction

Codencode Aligner (http://www.codoncode.com/aligner/) was used for sequence assembly and annotation. Protein-coding (PCGs) and rRNA genes were identified by sequence alignment [25] with published mitogenomes of other whiteflies. Both the base composition and codon usage were further analyzed with MEGA v. 5.0 [26].

Most tRNA genes were identified by tRNAscan-SE Search Server v. 1.21 (http://lowelab.ucsc.edu/tRNAscan-SE/) [27] using the default setting. The secondary structures of tRNA genes that could not be found by tRNAscan-SE were identified by comparison with other Hemipteran species. Secondary structures of small and large rRNA subunits were deduced based on models predicted for other species [28–31]. Strand asymmetry was calculated using the formulae: AT skew = [A−T]/[A+T] and GC skew = [G−C]/[G+C] [32]. The software packages DnaSP v. 5.10 [33] was used to calculate the synonymous substitution rate (Ks) and the nonsynonymous substitution rate (Ka). The tandem repeats within the putative control region were analyzed with the Tandem Repeats Finder program (http://tandem.bu.edu/trf/trf.advanced.submit.html) [34].

### Phylogenetic analysis

Ten complete Aleyrodidae mitogenomes were downloaded from GenBank to investigate phylogenetic relationships (Table A in S1 File). We chose *Sitobion avenae* and *Diuraphis noxia*, two Aphididae species, as outgroups. Thirteen PCGs were initially aligned with Clustal X, translated into amino acid sequences using default settings, and then analyzed with MEGA v. 5.0 [26]. Alignments of individual genes were concatenated, excluding the start and stop codons [25].

The best fit model for nucleotide alignments was determined by Modeltest v. 3.7 [35] using likelihood ratio tests. The GTR+I+G paradigm was considered to be the ideal model for phylogenetic analysis of amino acid sequence alignments. Based on Modeltest v. 3.7, phylogenetic trees were constructed using MrBayes v. 3.2.1 [36] and a PHYML online web server [37–38]. Bayesian inference (BI) analyses were processed with 3,000,000 generations and four chains (one cold and three hot chains), with sampling every 100 generations and a burnin of 25% [39–40]. The confidence values of the BI tree were shown as Bayesian posterior probabilities in percentages. Multiple genome arrangements (MGR) [41] was also used for constructing phylogenies based on gene orders of the eleven whitefly mitogenomes.

## Results and Discussion

### Genome organization and composition

**Fig 1:**
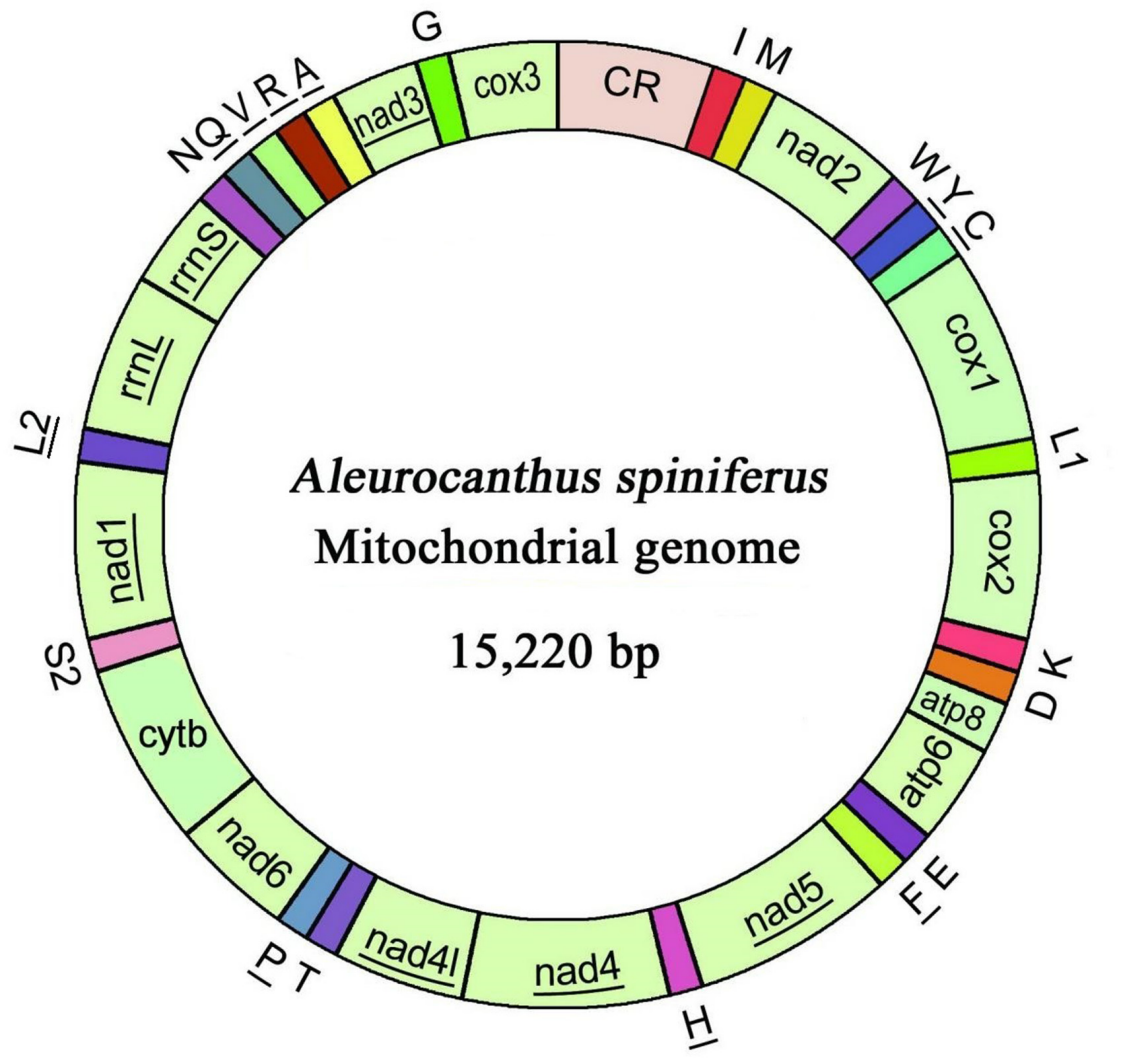
Mitochondrial map of *A*. *spiniferus*. Genes that are not underscored are transcribed on the majority strand (J-strand; an exception is the control region, CR). Genes that are underscored are transcribed on the minority strand (N-strand). tRNA genes are designated by single letter amino acid codes outside the map.

The complete mitogenome of *A*. *spiniferus* was a 15,220 bp circular DNA molecule (GenBank accession no. KJ437166) (Fig 1, Table 1). The mitogenome contained 36 genes, including 13 PCGs and two rRNA genes. The *A*. *spiniferus* mitogenome contained 21 tRNA genes instead of the typical 22 found in many other metazoan mitogenomes. There was also a large non-coding region (the CR). Substantial rearrangements and altered transcriptional directions were observed in the tRNAs, rRNAs and PCGs relative to other insect mitogenomes. Seventeen *A*. *spiniferus* genes were transcribed on the J-strand and the remaining 19 were transcribed on the N-strand.

**Table 1:** Gene structure of the mitogenome of *Aleurocanthus spiniferus*.

Gene	Position	Size	Direction^a^	IGN^b^	Anti- or start/	A+T
	(bp)	(bp)			stop codons^c^	content (%)
*cox1*	1–1539	1539	Forward	2	ATG, TAA	65.0
*trnL1* (*UUR*)	1542–1605	64	Forward	0	TAA	68.8
*cox2*	1606–2269	664	Forward	0	ATT, T--	69.0
*trnLys* (*K*)	2270–2339	70	Forward	6	CTT	72.8
*trnAsp* (*D*)	2346–2407	62	Forward	9	GTC	64.5
*atp8*	2417–2563	147	Forward	-1	ATA, TAA	63.3
*atp6*	2563–3207	645	Forward	18	ATA, TAG	68.6
*trnGlu* (*E*)	3226–3292	67	Forward	7	TTC	70.1
*trnPhe* (*F*)	3300–3362	63	Reverse	-20	GAA	77.8
*nad5*	3348–5022	1675	Reverse	0	ATA, T--	73.0
*trnHis* (*H*)	5023–5083	61	Reverse	1	GTG	80.3
*nad4*	5085–6365	1281	Reverse	-4	ATG, TAG	69.1
*nad4l*	6362–6658	297	Reverse	2	ATG, TAA	75.8
*trnThr* (*T*)	6661–6722	62	Forward	0	TGT	77.4
*trnPro* (*P*)	6723–6783	61	Reverse	25	TGG	73.7
*nad6*	6809–7249	441	Forward	-1	ATA, TAA	72.6
*cytb*	7249–8382	1134	Forward	0	ATG, TAA	66.2
*trnS2* (*UCN*)	8383–8439	57	Forward	-7	GCT	75.5
*nad1*	8433–9353	921	Reverse	0	ATA, TAA	72.5
*trnL2* (*CUN*)	9360–9422	63	Reverse	0	TAG	82.5
*rrnL*	9423–10700	1278	Reverse	5		77.7
*rrnS*	10706–11476	771	Reverse	9		77.1
*trnAsn* (*N*)	11486–11551	66	Forward	4	GTT	75.7
*trnGln* (*Q*)	11556–11626	71	Reverse	-7	TTG	88.8
*trnVal* (*V*)	11620–11683	64	Reverse	-3	TAC	73.4
*trnArg* (*R*)	11681–11745	65	Reverse	1	TCG	69.2
*trnAla* (*A*)	11747–11808	62	Reverse	0	TGC	75.8
*nad3*	11809–12161	353	Forward	1	ATT, T--	71.7
*trnGly* (*G*)	12163–12225	63	Forward	0	TCC	84.1
*cox3*	12226–13011	786	Forward	0	ATG, TAA	67.3
*CR*	13012–13933	920				65.9
*trnIle* (*I*)	13932–13997	66	Forward	-4	GAT	69.7
*trnMet* (*M*)	13994–14061	68	Forward	0	CAT	76.5
*nad2*	14062–15036	975	Forward	-2	ATA, TAA	68.5
*trnTrp* (*W*)	15035–15097	63	Forward	-2	TCA	80.9
*trnTyr* (*Y*)	15096–15157	62	Reverse	0	GCA	80.7
*trnCys* (*C*)	15158–15220	63	Reverse	1	GTA	92.1

^a^ Forward or reverse indicate that the gene is encoded by the J or N strand.

^b^ Intergenic nucleotide;

(-) indicates overlapping genes.

^c^ T--, represents incomplete stop codons.

The *A*. *spiniferus* mitogenome contained 51 bp of overlapping nucleotides; these spanned ten pairs of neighboring genes and ranged in length from 1 to 20 bp. The longest overlap (20 bp) existed between *trnE* and *trnF*. Overall, the complete mitogenome of *A*. *spiniferus* was very compact; the mitogenome contained only 91 bp of intergenic nucleotides (IGN). The IGNs were located between 14 pairs of neighboring genes and ranged from 1 to 25 bp; the longest IGN was a 25-bp region located between *trnT* and *trnP*.

The nucleotide base composition of the *A*. *spiniferus* mitogenome was as follows: A = 31.0%, T = 39.8%, C = 12.4%, G = 16.8%. The *A*. *spiniferus* mitogenome was significantly biased towards the A and T nucleotides (70.8%), and this percentage was lower than the A+T content of other whitefly mitogenomes. The overall A+T content of the PCGs was 69.2%; genes with the highest and lowest A+T content were *trnC* (92.1%), and *atp8* (63.3%). Furthermore, the AT-skew and GC-skew were calculated for the *A*. *spiniferus* mitogenome (Table B in S1 File). The strand bias of the *A*. *spiniferus* mitogenome was not consistent with the strand bias of metazoan mitogenome (positive AT-skew and negative GC-skew for the J-strand). The results showed that the AT-skew of the *A*. *spiniferus* mitogenome was -0.125 and was biased to use T rather than A; conversely, the GC-skew was 0.151 and was biased to use G rather than C.

### Protein-coding genes

The combined length of the 13 PCGs was 10,841 bp; the mean A+T content was 69.2% and ranged from 65% (*cox1*) to 75.8% (*nad4l*) (Table B in S1 File). Start and stop codons were confirmed by sequence alignment with corresponding genes obtained from other whiteflies. All PCGs initiated with ATN, six genes (*atp6*, *atp8*, *nad1*, *nad2*, *nad5 and nad6*) initiated with ATA, five with ATG (*cox1*, *cox3*, *nad4*, *nad4l* and *cytb*), and two with ATT (*cox2* and *nad3*) (Table 1). Although some PCGs in the order Hemiptera initiate with unusual initiation codons such as TTG or GTG, this was not the case for *A*. *spiniferus* or other whiteflies. Most of the 13 PCGs in *A*. *spiniferus* contained typical termination codons (e.g. TAA or TAG); exceptions included *nad3*, *cox2*, and *nad5*, which contained the incomplete termination codon T. It is important to note that the truncated termination codon T could be potentially completed through post-transcriptional polyadenylation [42].

**Fig 2:**
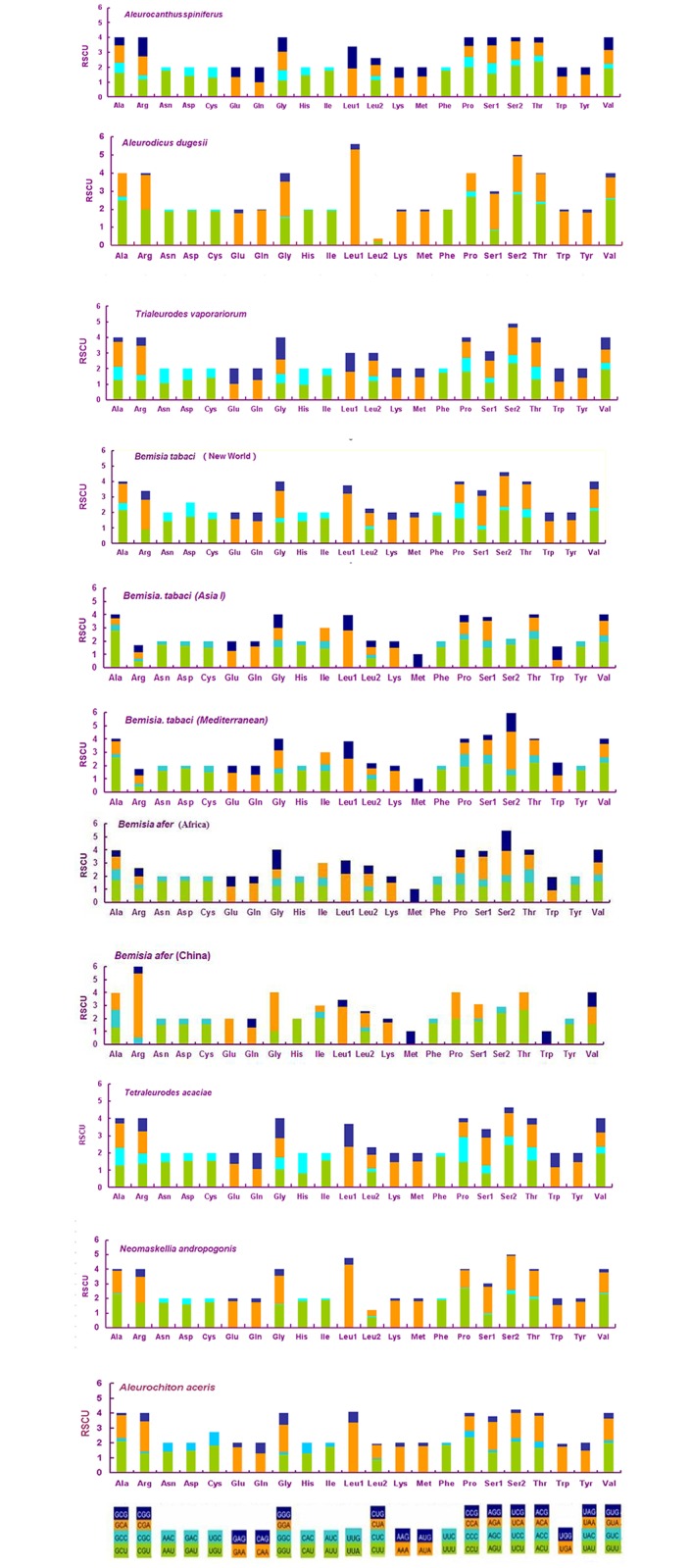
Relative synonymous codon usage (RSCU) in the mitogenomes of *A*. *spiniferus* and ten other species of Aleyrodidae. Codon families are given on the X-axis.

The base composition at each codon position of the concatenated PCGs was analyzed. The results showed that the A+T content of the first, second, and third codon positions was 68.8, 69.2, and 69.6%, respectively, and there was no obvious nucleotide bias. We summarized and compared the relative synonymous codon usage (RSCU) values of all eleven whiteflies in Fig 2, which reflected a biased usage of A and T nucleotides in all 11 species. The use of the anticodons NNA and NNU revealed the preference for A or T in the third position. Generally, the five most frequently used codons in *A*. *spiniferus* and the other ten whiteflies were TTT (Phe), ATT (Ile), TTA (Leu), ATA (Met) and AAT (Asn), which is consistent with the strong bias for A and T.

**Fig 3:**
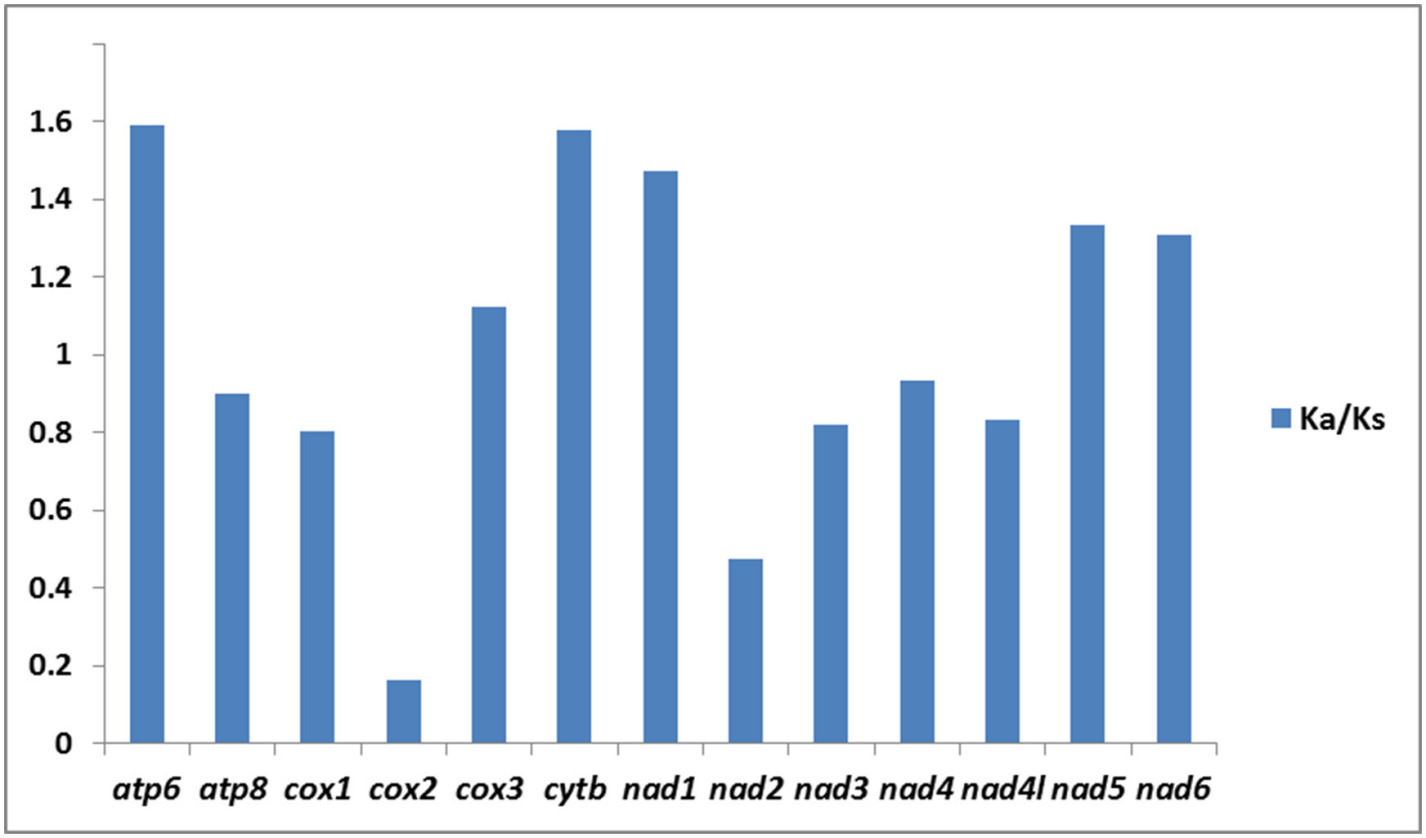
Evolutionary rates of PCGs in whitefly mitogenomes. The blue bar indicates the gene’s Ka/Ks.

In addition, the average ratio of Ka/Ks was calculated for each PCG of the 11 whitefly mitogenomes. The results showed that *atp6* had the highest evolutionary rate, followed by *cytb*, while *cox2* appeared to be the lowest (Fig 3). The ratios of Ka/Ks for *atp6*, *cox3*, *cytb*, *nad1*, *nad5* and *nad6* were above 1, indicating that these genes are evolving under positive selection. Simultaneously, ratios of Ka/Ks for other seven PCGs were all below 1, indicating the existence of purifying selection in these genes. Therefore, *cox2* and *nad2* with relatively slow rates may be candidate DNA barcoding markers. By contrast, *atp6*, *cytb* and *nad1* can be selected as an effective molecular marker to reconstruct evolutionary relationships at the species level and contribute to population studies of whiteflies.

### Transfer RNA genes

**Fig 4:**
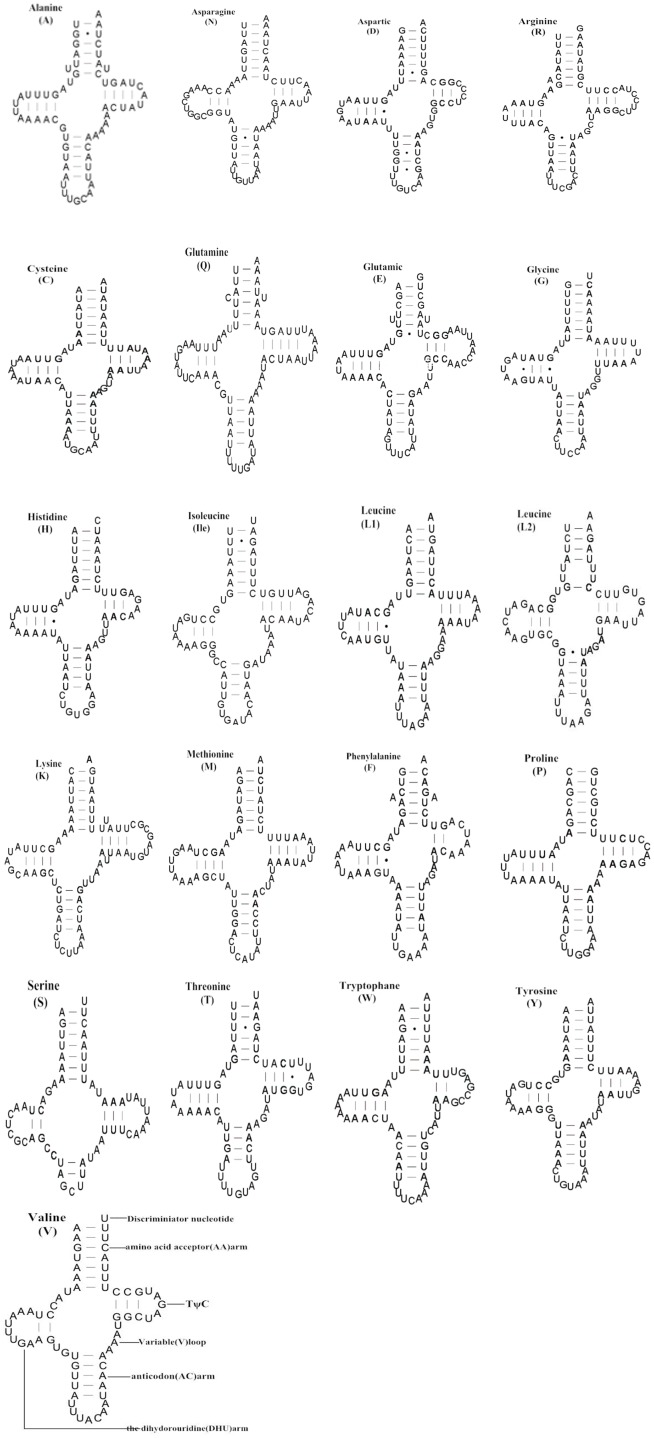
Inferred secondary structures of 21 tRNAs from the *A*. *spiniferus* mitogenome. The tRNAs are labelled with the abbreviations of their corresponding amino acids. Dashes (-) indicate Watson-Crick bonds and dots (·) represent GU bonds. Each arm and loop is illustrated for *trnV*.

The combined length of all tRNA genes was 1,343 bp; individual genes ranged from 57 to 71 bp with an average A+T content of 76.7% (Table A in S1 File). Unlike typical metazoan mitogenomes that contain 22 tRNA genes, the *A*. *spiniferus* mitogenome possessed 21 tRNA genes and lacked *trnS1* (AGN). With the exception of *trnS2* (UCN), the tRNAs were identified using tRNAscan-SE [27]. The secondary structure of *trnS2* was inferred by comparison with other Hemipteran mitogenomes. There were 15 mismatched nucleotides in the 21 tRNA genes of *A*. *spiniferus*, and these were all G-U pairs (Fig 4). All tRNA genes of *A*. *spiniferus* were predicted to fold into typical cloverleaf secondary structures (Fig 4). However, unlike other metazoan mitogenomes [43], the dihydrouridine (DHU) arms of *trnS2* in *A*. *spiniferus* formed a complete arm, not a D loop. Generally, the anticodons and secondary structures of the 21 tRNAs were essentially identical to those described in *Locusta migratoria* and *Liriomyza trifolii* [44–45].

### Ribosomal RNA genes

**Fig 5:**
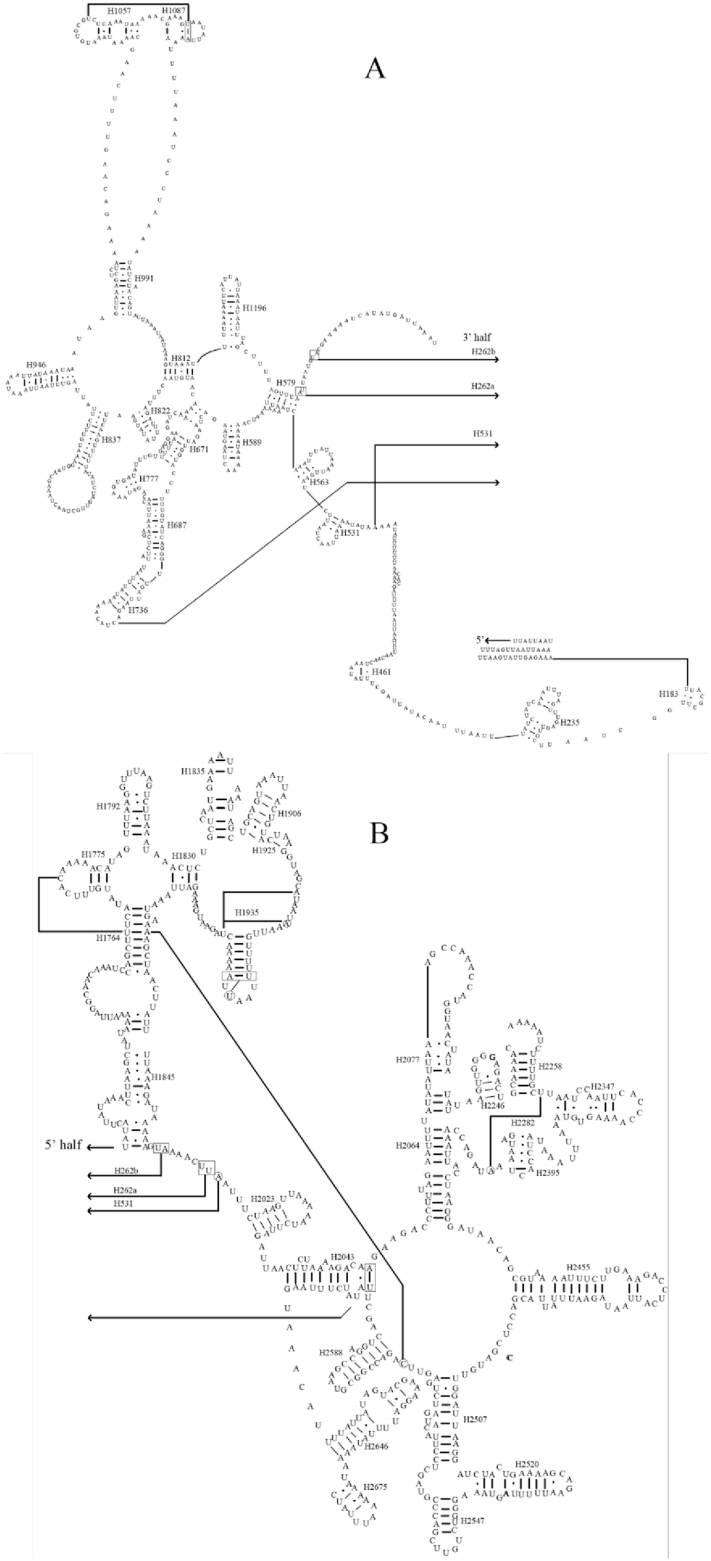
Predicted secondary structure of *rrnL* in the *A*. *spiniferus* mitogenome. Tertiary interactions and base triples are depicted with continuous lines. (A) represents the 5' end of *rrnL*, and the 3' portion is shown in (B). Base-pairing is indicated as follows: Watson-Crick pairs, lines; GU pairs, dots; other non-canonical pairs, circles.

**Fig 6:**
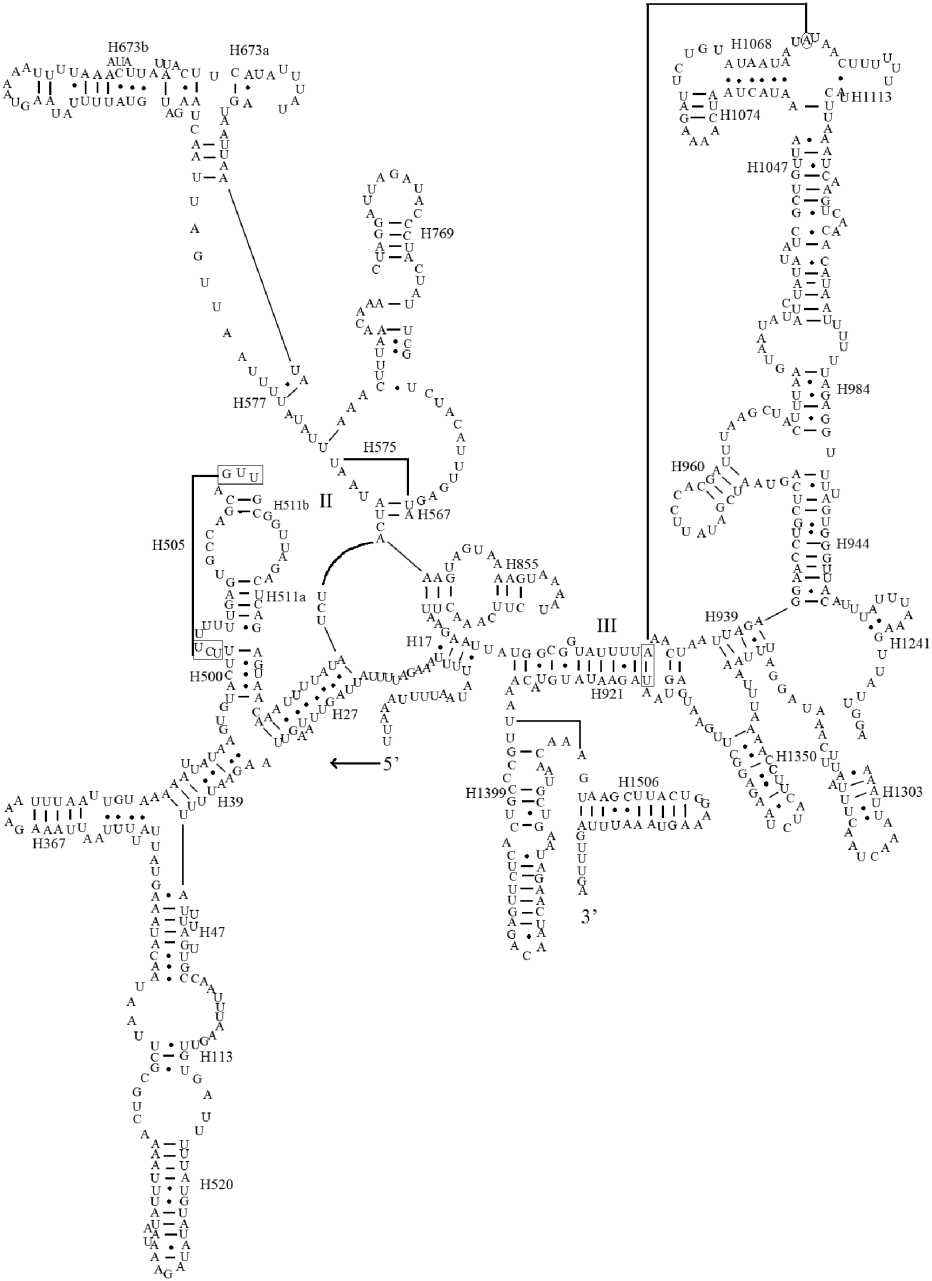
Predicted secondary structure of *rrnS* in the *A*. *spiniferus* mitogenome. Tertiary interactions and base triples are illustrated with continuous lines. Base-pairing is indicated as follows: Watson-Crick pairs, lines; GU pairs, dots; other non-canonical pairs, circles.

The large ribosomal RNA (*rrnL*) gene of *A*. *spiniferus* was 1,278 bp in length with an A+T content of 77.7%, and the small ribosomal RNA (*rrnS*) gene was 771 bp with an A+T content of 77.1%. *rrnL* and *rrnS* were located between *trnL2* (CUN) and *trnN* (Fig 1), presumably due to genetic rearrangements. In *A*. *spiniferus*, the two RNA genes were contiguous and not separated by *trnV*; the latter is common in other insect mitogenomes. The secondary structure of *A*. *spiniferus* rRNAs corresponded with models proposed for other insects [28–31]. The secondary structure of *rrnL* consisted of six domains (domain III is absent in arthropod mitogenomes) with 47 helices (Fig 5), whereas *rrnS* contained 31 helices belonging to three domains (Fig 6).

### The control region

**Fig 7:**
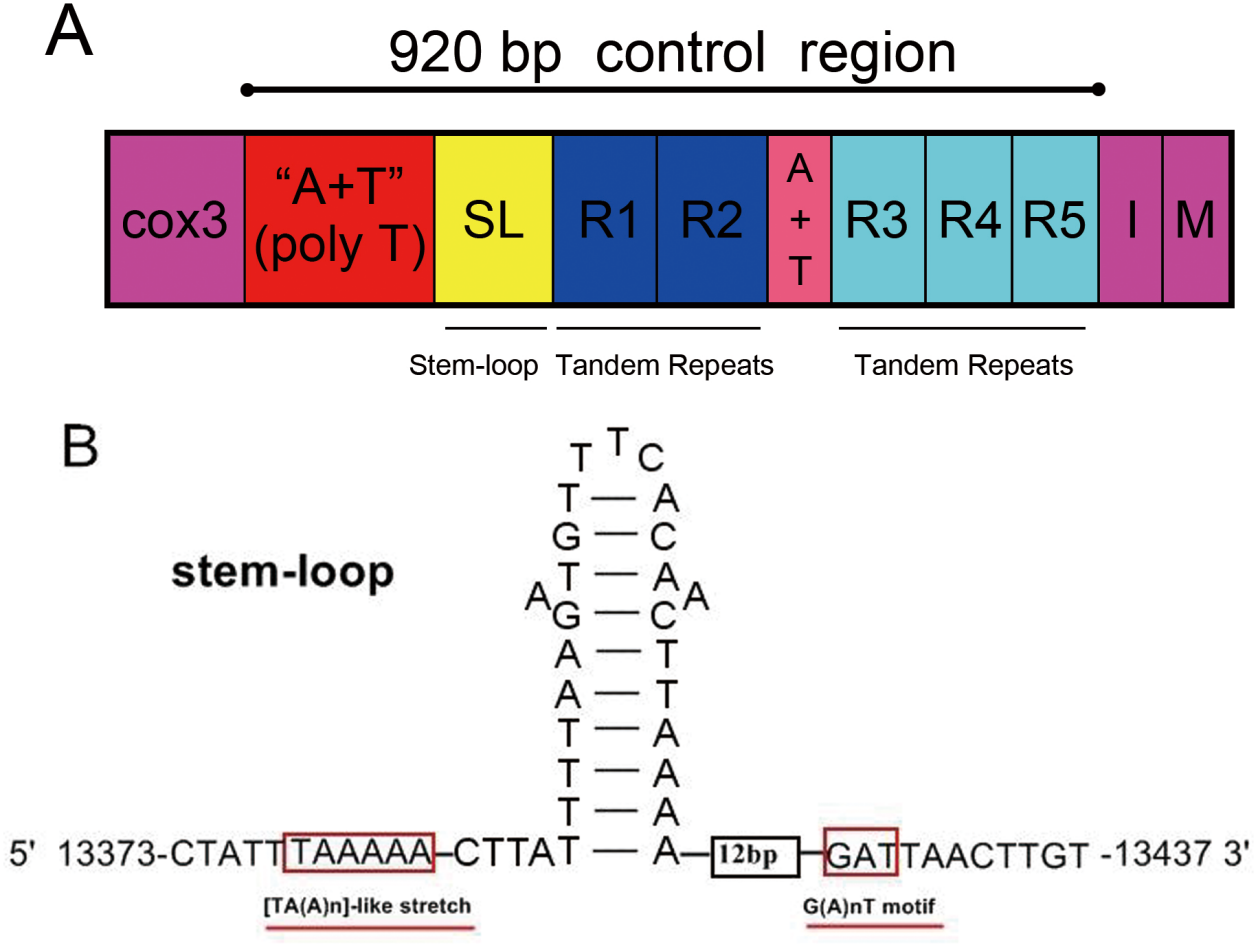
Predicted structural elements in the control region of *A*. *spiniferus*. (A) The control region flanking genes *cox3*, *trnI* (I) and *trnM* (M) is represented by a purple shaded rectangle. The A+T regions are indicated by red and pink shaded rectangles. The yellow shaded box indicates stem-loop regions; dark blue and aqua shaded boxes represent the five tandem repeats, R1-R5. (B) Putative stem-loop structures found in the control region. The red outlined boxes indicate highly-conserved flanking sequences.

In *A*. *spiniferus*, the large non-coding, control region (CR) contained 920 bp nucleotides, and the A+T content was 65.9%. The CR was located between the *nad3-trnG-cox3* and *trnI-trnM* gene clusters, and this unusual location for CR is presumably due to genetic rearrangement. The CR of *A*. *spiniferus* could be subdivided into five parts (Fig 7): (1) a 293-bp region where the G+C content (39%) was slightly higher than the mitogenome (29.2%); this was adjacent to 69-bp poly-T region; (2) a 63-bp segment, containing a 44-bp putative stem-loop structure; (3) a region containing two 128-bp tandem repeats (R1 and R2); (4) a 94-bp intervening region with an A+T content (56%) lower than the mitogenome (70.8%); this region contained a 71-bp segment that contained sequences conserved in the tandem repeats (R1 / R2); and (5) a region containing three 38-bp tandem repeats (R3, R4, and R5); an exception was R5, which had a 6-bp deletion.

There are very few reports that compare the CRs of different whitefly species. Wang et al. [46] compared the CRs from two biotypes of *Bemisia tabaci*, and each biotype had two putative CRs. Thus the CRs of whiteflies obviously vary in length, nucleotide content, and structure when compared with other insects. CR length is generally dictated by the number and length of tandem repeats. The CR of *A*. *spiniferus* contained a single stem-loop structure, which was probably associated with the initiation of mitogenome replication or transcription [47]. Two motifs in the stem-loop structure, TATA at the 5′ end and G(A)_n_T motif at the 3′ end of the stem-loop structure(s), are conserved in some insect species [48]. However, the [TA(A)]_n_-like stretch in *A*. *spiniferus* was different from the 4-bp motif (TATA) observed in other whiteflies.

### Gene rearrangement

Genetic rearrangements in insect mitogenomes occur randomly between orders but are very informative with respect to insect evolution [49]. The first insect mitogenome to be sequenced was obtained from *Drosophila yakuba*; this was regarded as the model mitogenome because of its conserved gene order when compared with non-insect hexapods [50] and crustaceans [51]. The *Aleurodicus dugesii* mitogenome is similar to *D*. *yakuba* and is recognized as the model mitogenome of whiteflies [13, 52].

**Fig 8:**
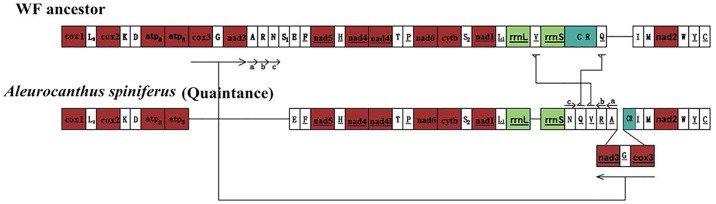
Comparisons of mitogenome arrangements between the presumed ancestral whitefly and *A*. *spiniferus*. Genes are transcribed from left to right except for the underscored genes that are transcribed in the opposite direction. Horizontal bars between genes indicate that they are contiguous with few (if any) nucleotides gaps. The principal changes are indicated by thick lines with arrowheads; the orientation of arrowheads shows the direction of transcription.

We noted extensive variation in the *A*. *spiniferus* mitogenome when compared to the *D*. *yakuba* mitogenome. In this respect, our findings are similar with those reported previously [13]. Thao et al. sequenced the mitogenomes of six whitefly species and found that four species showed genetic rearrangements and lacked one or more tRNA genes [13]. The rearrangements observed in the *A*. *spiniferus* mitogenome were similar to the A type observed in *B*. *tabaci* [13]. The *cox3-trnG-nad3* ancestral gene cluster was rearranged in *A*. *spiniferus* and occurred in the reverse order (*nad3-trnG-cox3*) between *trnA* and CR. The ancestral cluster consisting of *trnA-trnR-trnN* was translocated in *A*. *spiniferus* and mapped between *rrnS* and the *nad3-trnG-cox3* gene cluster. In addition, *trnS1* (AGN) was absent from the *A*. *spiniferus* mitogenome, and *trnV* and *trnQ* were placed between *trnN* and *trnR* (Fig 8). The rationale and mechanisms underlying these rearrangements in the *A*. *spiniferus* mitogenome are unclear; nonetheless, these results greatly contribute to our knowledge of whitefly mitogenome phylogeny.

**Fig 9:**
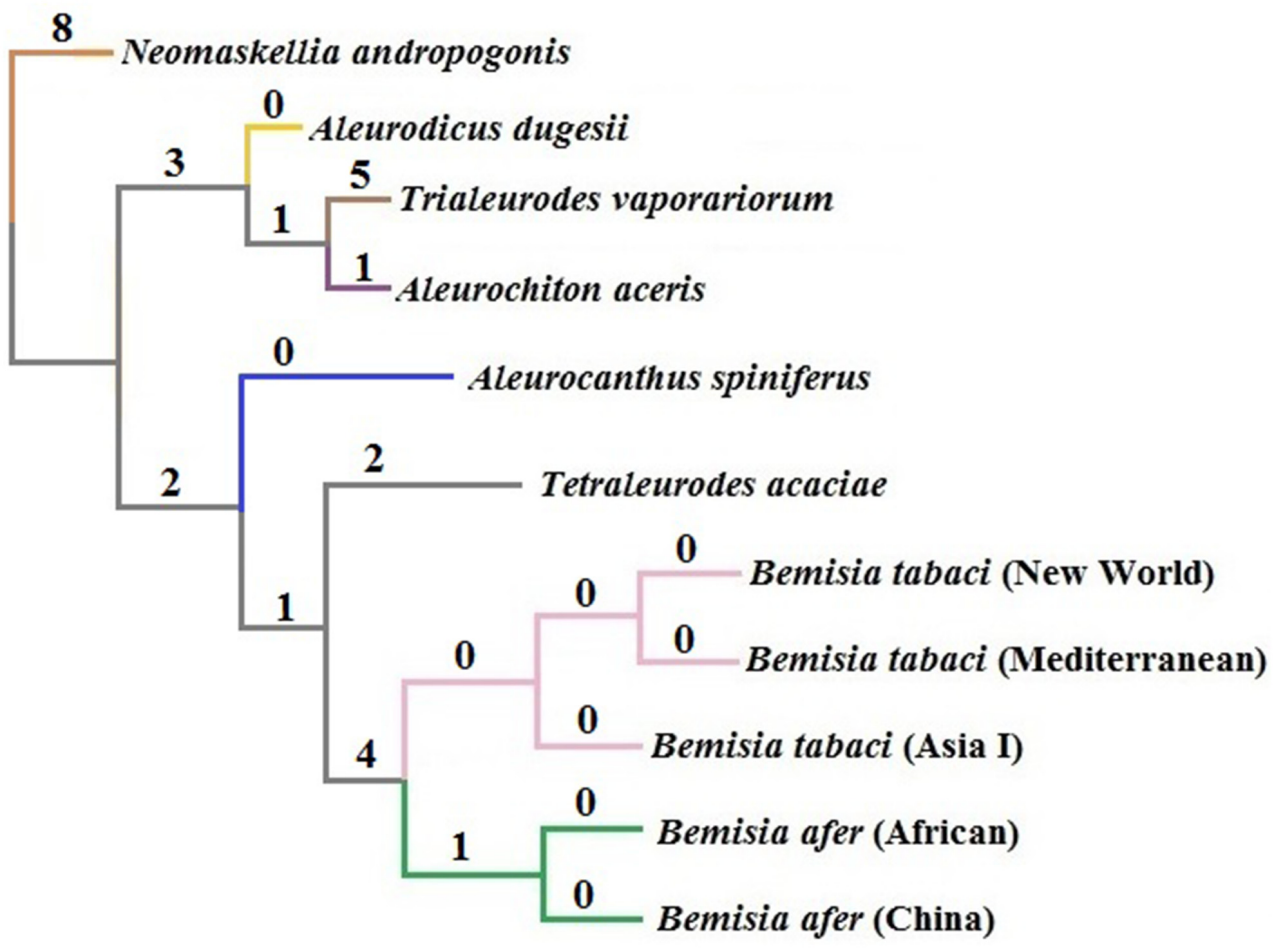
Phylogeny of 11 whitefly mitogenomes reconstructed by MGR. The number of rearrangements is shown numerically on the branches.

**Fig 10:**
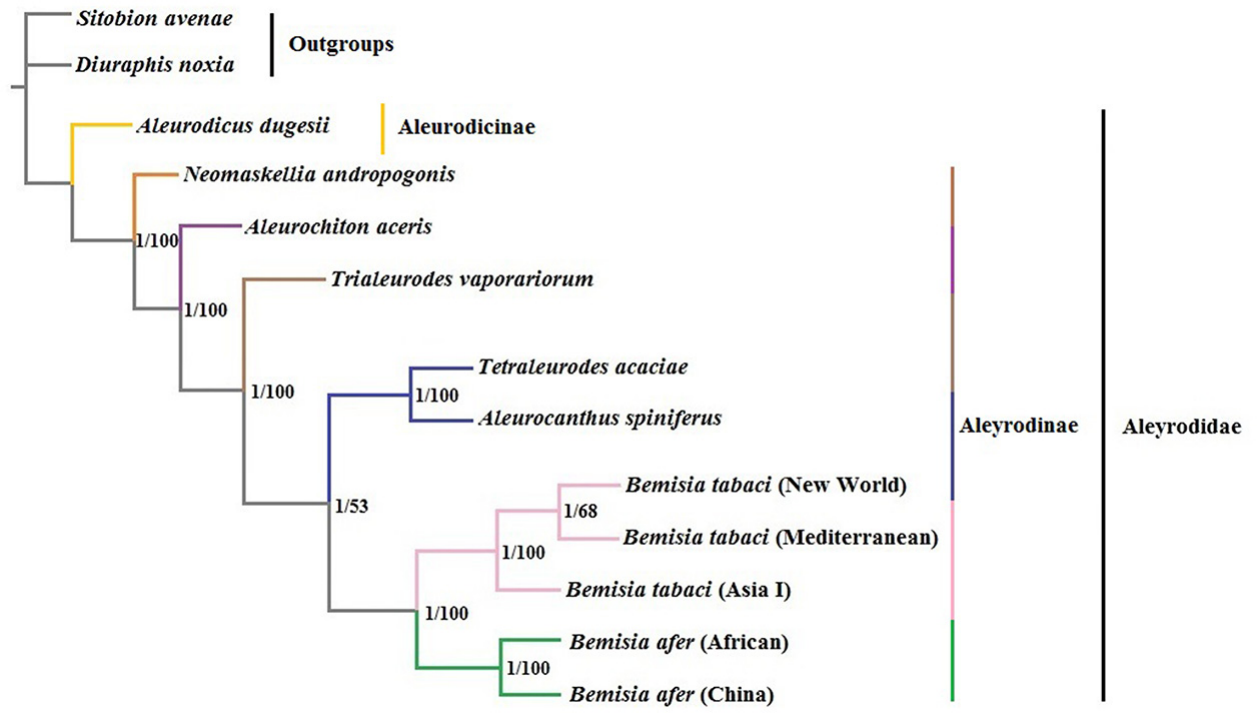
Inferred phylogenetic relationships among Aleyrodidae based on the 13 PCGs. Numbers at the nodes are Bayesian posterior probabilities (left) and ML bootstrap values (right). The tree was rooted with two outgroups (*S*. *avenae* and *D*. *noxia*).

To date, 11 whitefly mitogenomes have been sequenced, and nine exhibit rearrangements that indicate translocation of the *nad3-trnG-cox3* gene cluster [13, 46, 53–54]. Furthermore, the absence of one or more tRNAs has been documented in *Neomaskellia andropogonis*, *Aleurochiton aceris*, *Aleurodicus dugesii*, *T*. *acaciae*, *A*. *spiniferus* (this study) and *Bemisia afer* (Africa). The sequences of additional whitefly mitogenomes are needed to confirm whether the translocation of the *nad3-trnG-cox3* gene cluster is a common feature in the Aleyrodidae. It is also noteworthy that the phylogenetic tree recovered by MGR is very similar to the trees constructed by BI and ML, which shows that a total of 28 rearrangements occurred in the published mitogenomes of Aleyrodidae (Figs 9 and 10).

### Phylogenetic characteristics and evolution of whiteflies

The analysis of PCGs encoded by the mitogenomes has emerged as an informative strategy for inferring phylogenetic relationships [55]. In our study, phylogenetic analyses were conducted based on a concatenated nucleotide data set containing 13 PCGs from 11 Aleyrodidae and two Aphididae species (the latter were included as outgroups; Table A in S1 File). Furthermore, the two phylogenetic trees generated by BI and ML analyses were identical (Fig 10). Both analyses supported the division of the Aleyrodidae into subfamilies Aleurodicinae and Aleyrodinae. *A*. *dugesii* belonged to Aleurodicinae. In the Aleyrodinae, our results support the assignment of *A*. *spiniferus* as a sister group to *T*. *acacia*. The three *B*. *tabaci* species grouped together as did the two *B*. *afer* spp. These results were consistent with morphologic classification and phylogenetic studies of whiteflies [13, 56–59]. Notably, the phylogeny of the *B*. *tabaci* complex supported the close relationship between New World and Mediterranean species, which was observed by Lee et al. [57] and Boykin et al. [60]. However, De Barro et al. [61] and Dinsdale et al. [62] reported that the New World species were closer to the Asia I than the Mediterranean species. In an earlier study conducted by Boykin et al. [58], the phylogenetic relationship between the three *B*. *tabaci* groups was variable.

**Fig 11:**
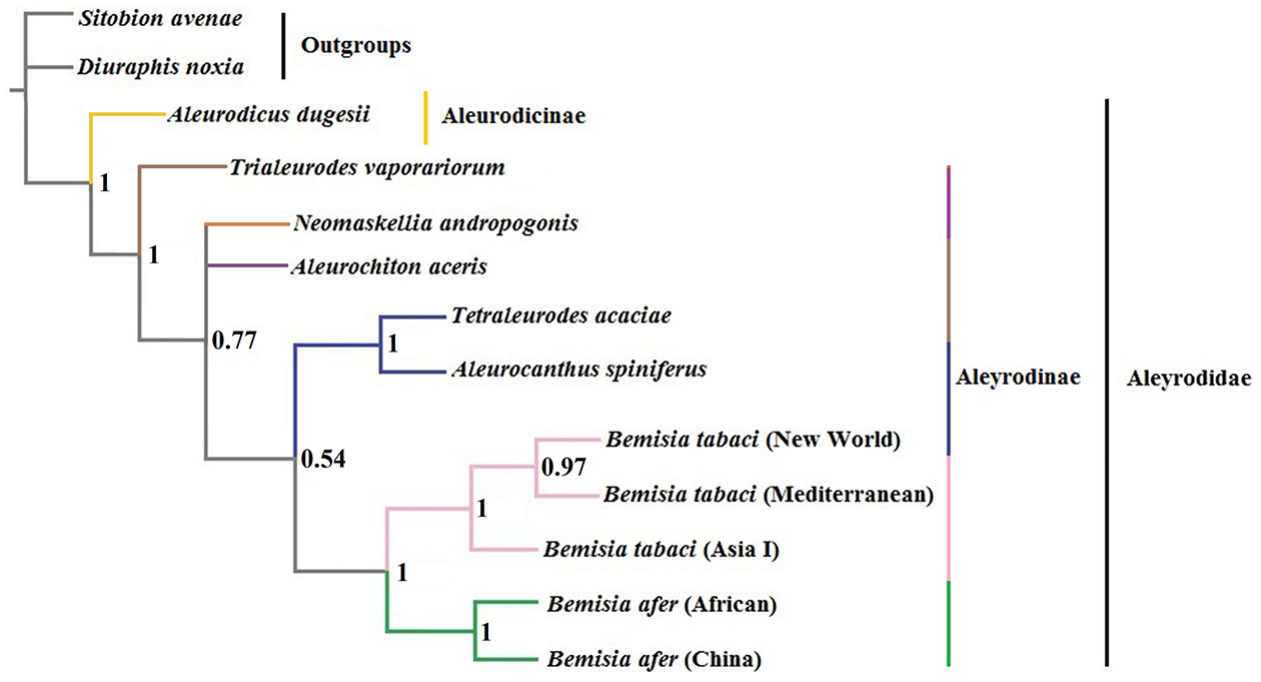
Inferred phylogenetic relationships among Aleyrodidae based on three selected PCGs. Numbers at the nodes are Bayesian posterior probabilities. The tree was rooted with two outgroups (*S*. *avenae* and *D*. *noxia*).

Based on three selected PCGs (*atp6*, *cytb* and *nad1*) with high evolutionary rates, an additional phylogenetic tree was constructed by BI analysis (Fig 11). Topology of this tree was similar to the former one in Fig 9, except that the relative position of *N*. *andropogonis*, *A*. *aceris* and *Trialeurodes vaporariorum* was changed. The inconsistency in these studies may be due to the different molecular data and models selected for analyses.

Analysis of Arthropoda indicated that species with high rates of genetic rearrangement tended to have high evolutionary rates [63]. Our phylogenetic analysis of the Aleyrodidae agreed with the conclusion of Thao et al. [13]; namely, within the Aleyrodidae, species with relatively conserved mitogenomes often group at the base of the phylogenetic tree, while species with higher rates of genetic rearrangement often occupy a more evolved position (Figs 9 and 10). These phenomena suggest that the rearrangement of mitogenomes were heightened during evolution in Aleyrodidae. However, additional molecular data and subsequent analyses are clearly needed to clarify the genetic arrangements and evolutionary characteristics within the Aleyrodidae.

## Supporting Information

S1 File (DOCX)
