# Supplementary material for: Complete Mitochondrial Genome of the Citrus Spiny Whitefly Aleurocanthus spiniferus (Quaintance) (Hemiptera: Aleyrodidae): Implications for the Phylogeny of Whiteflies
Source: PLoS One. 2016 Aug 23;11(8):e0161385. doi: 10.1371/journal.pone.0161385 (PMC4995055; doi:10.1371/journal.pone.0161385)
Supplement: S1 File — (DOCX) [file pone.0161385.s001.docx]

**Table A. Summary of mitogenome sequences used in present study.**

| **Family** | **Species** | **GenBank Accession No.** | **Reference** |
| --- | --- | --- | --- |
| Aphididae | [Diuraphis noxia](http://www.ncbi.nlm.nih.gov/Taxonomy/Browser/wwwtax.cgi?lvl=0&id=143948) | [NC_022727](http://www.ncbi.nlm.nih.gov/nuccore/556506243) | Thao et al. (2004) |
| Aleyrodinae | *Aleurochiton aceris* | [NC_006160](http://www.ncbi.nlm.nih.gov/nuccore/51830142) | Thao et al. (2004) |
|  | *Bemisia tabaci* | [NC_006279](http://www.ncbi.nlm.nih.gov/nuccore/52220940) | Thao et al. (2004) |
|  | (New World) |  |  |
|  | *Bemisia tabaci* | JQ906700 | Wang et al. (2013) |
|  | (Mediterranean) |  |  |
|  | *Bemisia tabaci* | KJ778614 | Tay et al. (2014) |
|  | (Asia I) |  |  |
|  | *Bemisia afer* | KF734668 | Wang et al. (2014) |
|  | (African) |  |  |
|  | *Bemisia afer* | KR819174 | Wang et al. (2015) |
|  | (China) |  |  |
|  | *Neomaskellia andropogonis* | [NC_006159](http://www.ncbi.nlm.nih.gov/nuccore/51830183) | Thao et al. (2004) |
|  | *Tetraleurodes acaciae* | [NC_006292](http://www.ncbi.nlm.nih.gov/nuccore/52221066) | Thao et al. (2004) |
|  | *Trialeurodes vaporariorum* | [NC_006280](http://www.ncbi.nlm.nih.gov/nuccore/52220968) | Thao et al. (2004) |
| Aleurodicinae | *Aleurodicus dugesii* | [NC_005939](http://www.ncbi.nlm.nih.gov/nuccore/49146478) | Thao et al. (2004) |

**Table B. Nucleotide composition of the *Aleurocanthus spiniferus* mitogenome.**

| Feature | A% | T% | C% | G% | A+T% | AT- | GC- | No. of nucleotides |
| --- | --- | --- | --- | --- | --- | --- | --- | --- |
|  |  |  |  |  |  | Skew | Skew |  |
| Whole mitogenome | 31.0 | 39.8 | 12.4 | 16.8 | 70.8 | -0.125 | 0.151 | 15220.0 |
| Protein-coding genes | 29.2 | 40.0 | 12.5 | 18.3 | 69.2 | 0.156 | 0.189 | 10841.0 |
| 1st codon position | 27.3 | 42.0 | 12.7 | 18.5 | 68.8 | 0.208 | 0.184 | 3613.0 |
| 2nd codon positon | 30.3 | 39.0 | 11.8 | 18.9 | 69.2 | 0.124 | 0.230 | 3614.0 |
| 3rd codon position | 30.1 | 40.0 | 12.9 | 17.5 | 69.6 | 0.135 | 0.152 | 3614.0 |
| Protein-coding genes-J | 23.4 | 44.0 | 12.5 | 20.2 | 67.3 | -0.306 | 0.235 | 5544.0 |
| 1st codon position | 25.8 | 39.0 | 12.1 | 23.4 | 64.5 | -0.200 | 0.317 | 1848.0 |
| 2nd codon positon | 21.9 | 43.0 | 14.3 | 20.8 | 64.9 | -0.325 | 0.185 | 1848.0 |
| 3rd codon position | 22.3 | 50.0 | 11.1 | 16.4 | 72.5 | -0.384 | 0.193 | 1848.0 |
| Protein-coding genes-N | 35.4 | 35.8 | 12.5 | 16.3 | 71.21 | -0.006 | 0.244 | 5297.0 |
| 1st codon position | 39.1 | 35.0 | 9.3 | 17.0 | 73.7 | 0.059 | 0.086 | 1766.0 |
| 2nd codon positon | 38.3 | 28.0 | 14.7 | 18.6 | 66.6 | 0.148 | 0.158 | 1766.0 |
| 3rd codon position | 28.8 | 44.0 | 13.4 | 13.4 | 73.3 | -0.214 | 0.5 | 1765.0 |
| tRNA genes | 36.9 | 39.8 | 11.8 | 11.5 | 76.7 | -0.039 | -0.010 | 1343.0 |
| tRNA genes-J | 36.3 | 36.9 | 12.4 | 14.4 | 73.2 | -0.08 | 0.075 | 645.0 |
| tRNA genes-N | 37.4 | 42.6 | 11.2 | 8.9 | 79.0 | -0.065 | -0.114 | 698.0 |
| rRNA genes | 36.6 | 40.9 | 11.5 | 11.0 | 77.5 | -0.195 | -0.021 | 2049.0 |
| Control region | 31.4 | 34.5 | 14.3 | 19.8 | 65.9 | -0.046 | 0.159 | 920.0 |
